# Gender differential secular trend in lifetime smoking prevalence among adolescents: an age-period-cohort analysis

**DOI:** 10.1186/s12889-019-7735-8

**Published:** 2019-10-25

**Authors:** Jun Hyun Hwang, Soon-Woo Park

**Affiliations:** Department of Preventive Medicine, Catholic University of Daegu School of Medicine, 3056-6 Daemyung-4Dong, Nam-gu, Daegu, 705-718 Republic of Korea

**Keywords:** Age-period-cohort analysis, Cohort effect, Secular trend, Smoking prevalence

## Abstract

**Background:**

There has been a gender difference in adolescents’ lifetime smoking prevalence trends over the last 10 years. This study aimed to explain the gender differential secular trend in adolescents’ lifetime smoking prevalence using age-period-cohort (APC) analysis and suggests possible causes for this trend, including Korean tobacco control policies during the last 10 years.

**Methods:**

We utilized the 2006–2017 Korea Youth Risk Behavior Web-based Survey enrolling grades 7 to 12. Using year of survey and year of entry into middle school, we classified 859,814 students who had ever smoked into 6 age groups, 12 periods, and 17 school admission cohorts. Using APC analysis with the intrinsic estimator method, the effects of age, period, and school admission cohort on lifetime smoking prevalence were analyzed according to gender.

**Results:**

Overall, there was a similar tendency of all the three effects on lifetime smoking prevalence between genders: an increasing age effect with grade, negative period effect with survey period, and similar pattern of school admission cohort groups. However, compared to boys, girls experienced reduction in the increasing age effect in the 12th grade, consistent and steeper decreasing trend in the period effect from 2006 to 2016, and shorter and lower school admission cohort effect.

**Conclusions:**

Gender differential response to chronological changes in lifetime smoking prevalence was measured by the APC effect, which affected the gender differential secular trend in lifetime smoking prevalence. Therefore, considering the APC effect could help us understand the trend in smoking rates, as well as the contextual factors that affect it.

## Background

The Korean Youth Health Risk Behavior Web-based Survey (KYRBS) is a large-scale investigation conducted among 70,000 adolescents annually since 2005 on the level of health behavior of Korean adolescents. Ten years’ worth of Korean adolescent smoking data showed differences in trend patterns for the two genders (males: a slightly inverted U-shaped pattern, females: a continuously decreasing pattern) [1]. Simple studies on secular trends were conducted based on changes in smoking rates in different years [2, 3]. However, no studies have attempted to analyze the cause of gender-related smoking patterns or to define the changes in smoking rates among the cohort groups based on gender.

**Fig. 1:**
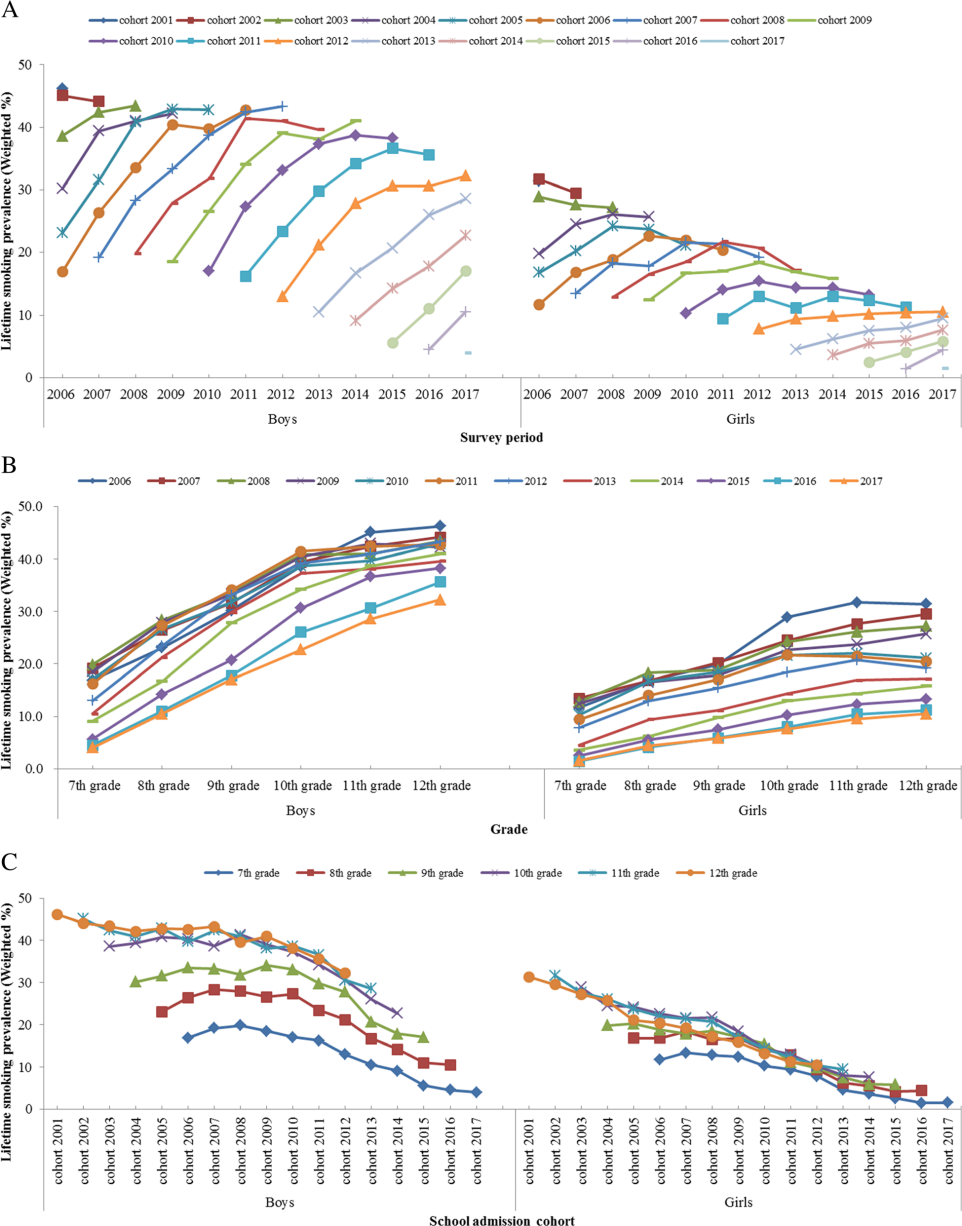
Lifetime smoking prevalence among boys and girls, by school admission cohort group (**a**), by survey period (**b**), and by grade group (**c**)

Although the KYRBS is a cross-sectional study, its representativeness and indicator characteristics (lifetime experience rate) show that in the school admission cohort group, the lifetime smoking rate theoretically cannot decrease as grade level increases. From this perspective, gender differences in smoking between school admission cohort groups show that although the male lifetime smoking rate does not decrease with increasing grade level, the female lifetime smoking rate has been shown to decrease with increasing grade level, after excluding parts of the cohort groups (Fig. 1a) [1].

When the cohort effect is non-existent or negligible in secular trend analysis, simple annual change could be evaluated. However, when it is considered that a cohort effect exists, like in the Korean adolescent lifetime smoking rate, age effect, period effect, and cohort effect should be considered for the cohort analysis of the secular trend [4]. Using age-period-cohort (APC) analysis, which was developed to explain the APC effect, smoking trend analysis in adults was conducted in USA [5, 6], England [7], Canada [8], Sweden [9], Korea [10], and Japan [11].

Tobacco control policies in Korea, including tobacco price increase, pictorial warning labels on cigarette packages, and extension of the smoking ban have been constantly reinforced during last 10 years [12]. As a result, tobacco policies may have affected the trend in Korean smoking prevalence either directly or through changes in social norms. Adolescents are more sensitive to tobacco control policies and social norms compared to adults [13]. The change in smoking rate could be drastic in a 1 year period. Hence, APC analysis could be helpful in understanding the secular trend. Two studies conducted by Chen et al. which evaluated the APC effect of smoking trend among adolescents showed that the cohort effect has caused a decrease in lifetime smoking prevalence among adolescents [14, 15]. However, many years of data from each birth cohort group are necessary for APC analysis, which is difficult to obtain from adolescents. The American Youth Risk Behavior Survey is conducted once every 2 years on 4 grade levels (9th~12th grade). Therefore, smoking rate information can only be obtained twice from the same cohort group [16]. In Korea, however, the KYRBS has been conducted annually for over 10 years at 6 different grade levels (7th - 12th grade), and sufficient data for APC analysis have been gathered for each cohort group.

Therefore, the purposes of this study were 1) to identify gender differences in the secular trend in Korean adolescents’ lifetime smoking prevalence from the APC effect perspective and 2) to suggest possible causes for this trend, including Korean tobacco control policies during last 10 years.

## Methods

### Study population

Data were obtained from 12 waves of national survey data: 12 KYRBS continuous surveys from 2006 to 2017 conducted by the Korea Centers for Disease Control and Prevention. There was no methodological change and the annual response rate was approximately 95% during this survey period. The KYRBS is a nationally representative, self-reported, and anonymous online survey of Korean students enrolled in grades 7 to 12. The KYRBS uses a stratified multistage probability sampling design to produce nationally representative statistics on adolescents’ health behaviors in Korea. A total of about 70,000 students from about 800 schools (400 middle schools and 400 high schools) have participated in the KYRBS [1]. Among survey participants of 12 waves of survey, a total of 859,814 (boys 443,800, girls 416,014) were analyzed. This secondary data analysis was approved as exempt from review by the Institutional Review Board of the Daegu Catholic University Medical Center (CR-18-015).

### Measures

Lifetime cigarette use is a reliable indicator with a concordance as high as 0.99 in the reliability test [17], and stable results can be obtained regardless of survey period or the smoking frequency unlike the measure of smoking over the past 30 days [14]. Therefore, we used the lifetime cigarette use in secular trend analysis that was determined by a self-reported response “yes” to the question “Have you ever smoked a cigarette, even one or two puffs?” This classification method for lifetime cigarette use was also used in previous studies using the Youth Risk Behavior Survey and the National Survey on Drug Use and Health [15, 18].

### Statistical analysis

Weighted lifetime smoking prevalence was calculated according to grade, period and school admission cohort via a complex sampling procedure using SPSS version 19.0 (IBM Corp., Armonk, NY, USA). APC analyses were used to identify the grade, period, and school admission cohort effects of lifetime smoking prevalence. For the APC analysis, the grade, period, and school admission cohort groups were divided into one-year intervals using 12 batches of data from KYRBS (2006–2017). As a result, we created 6 grade groups (7th grade to 12th grade), 12 period years (2006–2017), and 17 school admission cohorts (2001–2017).

To overcome the identification problem caused by the linear dependency among age, period, and cohort (cohort = period-cohort) in the APC analysis, the intrinsic estimator method based on principal components regression was developed [4]. Thus, we applied the intrinsic estimator method using the “apc_ie” command of STATA 13.0 (Stata Corp., College Station, TX, USA). The fitness of the APC model with possible combinations of age, period and cohort effects was analyzed using the Akaike information criterion and Bayesian information criterion. All analyses were performed separately for boys and girls.

## Results

Table 1 shows the gender-, grade-, and survey period-specific weighted lifetime smoking prevalences. The overall weighted lifetime smoking prevalence among boys increased until 2011 (from 32.5 to 34.3%) except for a slight decrease in 2010 but has declined since 2011 (from 34.3 to 20.1%). On the other hand, the overall lifetime smoking prevalence among girls has decreased steadily since 2006 (from 22.8 to 6.8%) (Table 1).

**Table 1:** Lifetime smoking prevalence according to gender, grade and survey period

Survey	Grade													
	7th		8th		9th		10th		11th		12th		Overall	
Period	N	Weighted%	N	Weighted%	N	Weighted%	N	Weighted%	N	Weighted%	N	Weighted%	N	Weighted%
Boys														
2006	6916	16.9	6404	23.1	6299	30.2	6039	38.6	6005	45.1	5540	46.2	37,203	32.5
2007	7102	19.2	6810	26.4	6709	31.6	6602	39.4	6199	42.4	6044	44.1	39,466	33.3
2008	6893	19.8	6938	28.3	6900	33.5	6674	40.8	6067	41.0	5806	43.4	39,278	34.2
2009	6933	18.5	6965	27.9	7035	33.3	6890	40.4	6104	42.9	5685	42.2	39,612	34.2
2010	6519	17.0	6620	26.6	6817	31.8	6229	38.7	6273	39.7	5933	42.8	38,391	32.9
2011	6548	16.2	6413	27.3	6589	34.1	6386	41.4	5954	42.4	5983	42.7	37,873	34.3
2012	6364	13.0	6394	23.4	6525	33.1	6606	39.1	6221	41.0	6111	43.3	38,221	32.5
2013	6411	10.5	6261	21.2	6249	29.8	6098	37.3	5595	38.1	6041	39.6	36,655	29.7
2014	6078	9.1	6331	16.7	6154	27.8	6048	34.2	6009	38.7	5850	41.0	36,470	28.4
2015	5576	5.6	6038	14.2	6244	20.7	5785	30.6	5777	36.6	5784	38.2	35,204	25.3
2016	5516	4.5	5466	11.0	5760	17.8	5861	26.0	5744	30.6	5456	35.6	33,803	21.9
2017	5178	4.0	5272	10.5	5202	17.0	5069	22.7	5610	28.6	5293	32.2	31,624	20.1
Girls														
2006	5960	11.7	5979	16.8	5861	19.8	5585	28.9	5445	31.7	5370	31.4	34,200	22.8
2007	5933	13.4	6039	16.8	6227	20.2	6207	24.5	5397	27.6	5429	29.5	35,232	21.7
2008	6144	12.8	6118	18.3	5950	18.8	6046	24.2	6308	26.1	5394	27.2	35,960	21.1
2009	5781	12.4	5903	16.5	5792	17.8	5587	22.6	6323	23.7	6068	25.7	35,454	19.7
2010	5949	10.3	5879	16.7	5786	18.5	5792	21.6	5851	22.0	5590	21.1	34,847	18.4
2011	6180	9.4	6490	14.0	6254	17.0	6183	21.7	6554	21.4	6109	20.4	37,770	17.3
2012	5998	7.8	5990	12.9	6026	15.4	5845	18.4	6094	20.7	6012	19.2	35,965	15.8
2013	5788	4.5	5852	9.4	5969	11.1	5930	14.3	6270	16.9	5971	17.1	35,780	12.3
2014	5583	3.6	5944	6.2	6066	9.8	5776	13.0	6143	14.3	6078	15.8	35,590	10.6
2015	5210	2.5	5404	5.5	5827	7.5	5337	10.2	5336	12.3	5725	13.2	32,839	8.8
2016	4967	1.4	5051	4.1	5459	5.9	5494	8.0	5326	10.4	5428	11.2	31,725	7.1
2017	5011	1.5	5105	4.4	5117	5.8	5096	7.6	5190	9.5	5133	10.5	30,652	6.8

On the whole, the weighted lifetime smoking prevalence increased with increasing grades in all survey periods. However, the weighted lifetime smoking prevalence in some senior grades (boys: 12th grade in 2009; girls: 12th grade in 2010, 2011 and 2012, and 11th grade in 2011) decreased compared to those in one grade below (Table 1, Fig. 1b).

Among the 15 school admission cohort groups surveyed for over 2 years (from 2002 to 2016 school admission cohort groups), the weighted lifetime smoking prevalence among boys increased with increasing grades in most school admission cohorts except in 5 cohort groups (2002, 2005, 2008, 2010, and 2011). However, among girls in all 10 early school admission cohort groups (from 2002 to 2011), the weighted lifetime smoking prevalence increased with increasing grade, but consistently declined in senior grades (10th – 12th grades) (Fig. 1a).

The weighted lifetime smoking prevalence by school admission cohort group in the same grade decreased in the late cohort groups. However, in the early cohort groups, increasing pattern of smoking prevalence until the mid-point of the survey period (from 2007 to 2010) among same grades were more pronounced among boys (Fig. 1c).

Table 2 shows the results of each of the seven models fitted. The full APC model, which had the lowest Akaike information criterion and Bayesian information criterion values, had the best fit for both boys and girls (Table 2).

**Table 2:** Goodness of fit statistics for age-period-cohort models for lifetime smoking prevalence among Korean adolescents

Model	Boys				Girls			
	df	Deviance	AIC	BIC	df	Deviance	AIC	BIC
Age	66	281,245.15	3919.53	280,962.90	66	498,388.68	6934.58	498,106.40
Period	60	894,526.41	12,437.49	894,269.80	60	328,835.35	4579.84	328,578.70
Cohort	55	422,229.42	5877.95	421,994.20	55	69,186.86	973.75	68,951.64
Age-period	55	82,418.03	1158.35	82,182.82	55	33,978.73	484.75	33,743.52
Age-cohort	50	14,920.01	221.02	14,706.18	50	15,548.14	228.91	15,334.30
Period-cohort	44	93,640.15	1314.52	93,451.97	44	33,849.43	483.26	33,661.26
Age-Period-Cohort	40	8077.92	126.27	7906.85	40	4977.51	82.37	4806.45

*df* Degree of freedom, *AIC* Akaike information criterion, *BIC* Bayesian information criterion

**Fig. 2:**
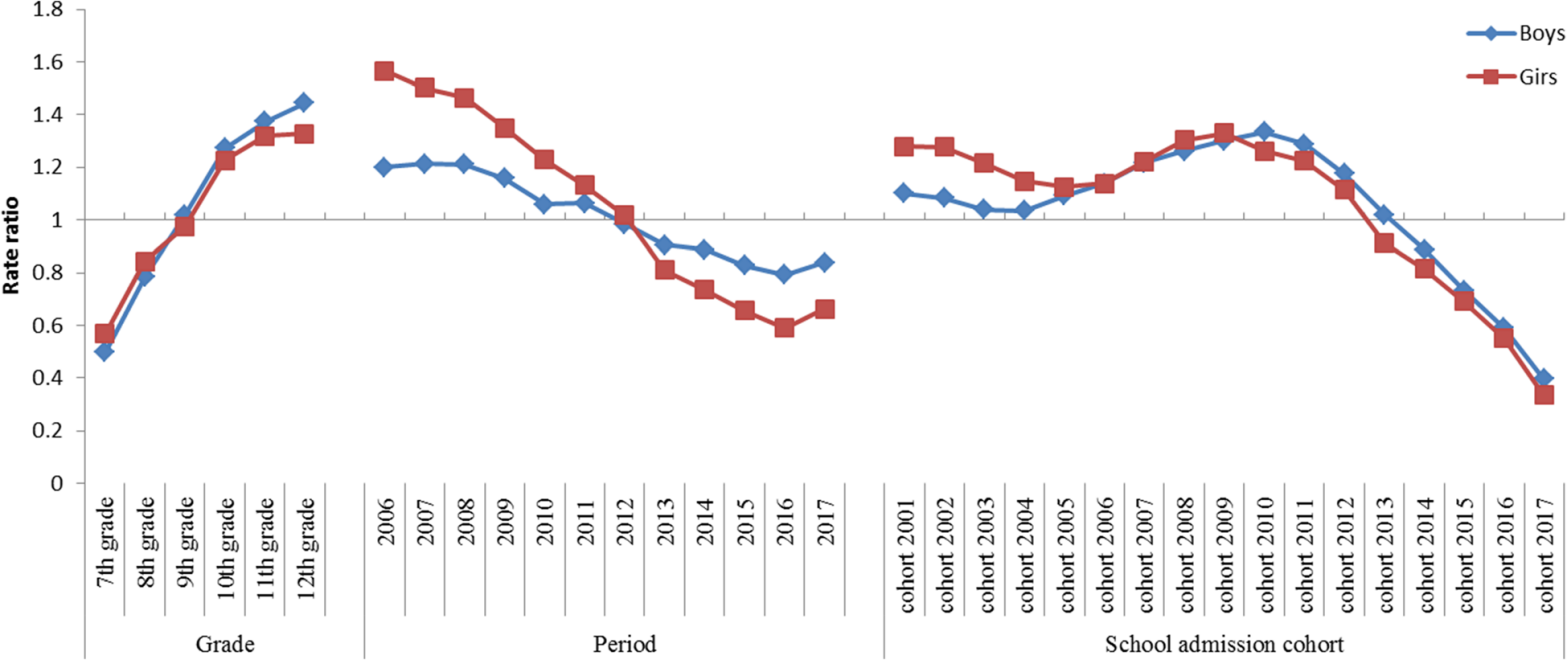
Age-period-cohort analysis of lifetime smoking prevalence among boys and girls

In Fig. 2, exponentiated coefficients (rate ratio) were plotted to show the net effects of age, period, and school admission cohort on lifetime smoking prevalence, and Additional file 1 shows the coefficient values of the APC effect. The age effect increased with grade and was the highest in the 12th grade in both sexes. The pattern of age effects from 7th grade to 11th grade was similar for both sexes, but the increasing change in age effect from 11th grade to 12th grade was relatively low in girls. The period effects were negatively associated with survey period from 2006 to 2016 except for the period 2007–2011 for boys, but rebound in 2017 compared to 2016 in both sexes. The slope change of coefficients of period effect in girls was steeper than that in boys. The pattern of the school admission cohort effect among girls was generally similar to that among boys, showing a decreasing pattern in early cohort groups (boys: 2001 to 2004, girls 2001 to 2005), increasing pattern in middle cohort groups (boys: 2004 to 2010, girls 2005 to 2009), and decreasing pattern in late cohort groups (boys: 2010 to 2017, girls 2009 to 2017). However, the duration and level of increasing cohort effect in middle cohort groups was slightly longer and higher in boys than in girls (coefficient estimates of school admission cohort effect: girls 2.44 times from 0.1171 in 2005 to 0.2855 in 2009, boys 7.35 times from 0.0393 in 2003 to 0.2888 in 2010) (Additional file 1).

## Discussion

In this study, an APC analysis was conducted to determine the lifetime smoking rate trends in each gender among Korean adolescents over the last 10 years from an APC effect perspective. The results showed that although no discernible directional difference in APC effect between the two genders was observed, detailed differences in the magnitude or duration of the APC effect have been observed as follows.

### Age effects

Although the age effect of the lifetime smoking rate increased with increasing grade level, the magnitude of increase was relatively small in higher grade levels (10th~12th grade). The decreasing age effect pattern was also observed in a study conducted by Chen et al., who used the data of never smoking prevalence among American adolescents between the ages of 13~17 years [14].

When lifetime smoking rate was compared between different grade levels within the same admission cohort group, decreased lifetime smoking rate at high grade levels (11th~12th grade) was consistently observed in the initial 10 admission cohorts of female students (2002 to 2011). Although a decrease in experience rate with increasing age cannot theoretically occur in a longitudinal study, it could be observed if representativeness in each grade is not guaranteed in admission cohort analysis using repeated cross-sectional surveys. However, the overall response rate was around 70% and the YRBS, which was conducted among about 15,000 American students, did not show decreased lifetime smoking rate with increasing grade level within the same school admission cohort in 10 years with the exception of one occasion (girls: 37.7% 10th grade in 2013, 36.3% 12th grade in 2015) [16, 19]. The KYRBS used in this study had a sample size of 70,000 adolescents and a response rate of 95%, which is greater than that of the YRBS [20]. Each year, 6000 adolescents of different genders at different grade levels are investigated to yield a stable lifetime smoking rate of above 10% in each gender and grade level. Hence, the KYRBS guarantees the representativeness of the participants [1]. However, a decrease in lifetime smoking rate at high level grades only among female students shows a possibility of measurement error and the following two reasons could be the causes of this observation.

First, lifetime smoking experience responds to past memories. Hence, recall bias could be more frequent among former smokers whose smoking dated back long periods or among ex-occasional smokers (the so-called lighter smoker bias) [21]. The mean age for first smoking experience did not largely differ between the two genders (boys, 12.8 years; girls, 13.1 years). However, female smokers had a lower frequency and amount of smoking [1]. Among these female students, progression to higher grade levels could imply a single smoking experience in the past or smaller smoking rates as non-smoking. Therefore, recall bias is relatively more likely. Secondly, adolescents at higher grade levels give fake responses to conform to social expectations. Late adolescence (age 15~19 years) is a period of increased interest in moral reasoning in terms of cognitive development [22]. Based on Kohlberg’s theory of moral development, the stage of socially conforming moral thinking (stage 4 in conventional morality level) increases and the previous stages (children’s behavior based on punishment avoidance (stage 1), rewards (stage 2), and good relations with others (stage 3)) are decreased [23]. In Asian countries, Confucianism culture gives a negative perspective on female smokers. Therefore, the smoking rate between males and females differ largely [24] and the self-stigma associated with smoking is also higher in females [25]. In Korea, social disapproval was especially high among 4 Asian nations (China, Thailand, Malaysia, and Korea) [26]. This strict social norm leads half of female adult and adolescent smokers to hide their smoking habits in surveys [27, 28]. As a result, the moral development process in Korean female students make them sensitive to social expectations and consequentially fake responses in surveys. Due to unintentional and intentional reasons such as those discussed above, the lifetime smoking rate could have decreased and the rate increase could have slowed among female students at high grade levels due to the age effect.

### Period effects

The period effect of the lifetime smoking rate showed a negative association from 2006 to 2016 except for the rates among male students in 2007 and 2011. The tobacco control policy in Korea has been constantly reinforced after ratifying the World Health Organization’s (WHO) Framework Convention on Tobacco Control in 2005. In particular, due to continuous amendments of the National Health Promotion Act since 2011, the tobacco control policies in Korea have been introduced or constantly enforced in terms of non-price control policies: strengthening the ban on tobacco advertising, promotion and sponsorship, complete ban on smoking indoors and in public places, strengthening the health warning label on cigarette packages and advertisement, restriction on indication of flavoring contained on the packages or advertisement, Quitline number for counseling services to quit smoking on the packages, and ban on misleading terms such as “mild,” “low tar,” and “light” [12, 29]. These reinforcements of tobacco control policies prevent or delay the first smoking experience of never-smokers, which could have consistently affected the periodic effect pattern of the lifetime smoking rate. Particularly, the tobacco price increase at the end of 2015 (from 2500 won to 4500 won per cigarette pack) led to the strongest period effect in the negative direction in 2016. Despite the introduction of pictorial health warning labels on cigarette packs in December 2016, the magnitude of the period effect in 2017 returned to the levels in 2015. Although several tobacco control policies in 2006~2017 led to decreased lifetime smoking rate among adolescents, increased tobacco price had the single largest period effect on adolescents’ smoking experience.

Reinforcement of tobacco control policies creates a social pressure to stop smoking [30], which leads to widespread smoking denormalization perception. In Korea, where the society is not very lenient with female smokers, changes in social perspective on smoking leads to a relatively larger effect on female students. Hence, the period effect of lifetime smoking rate is expected to be larger among female students than among male students. The Global Youth Tobacco Survey conducted in Korea in 2005 and 2013 showed a decrease in susceptibility (‘never tobacco users’ susceptible to tobacco use in the future, accessed by response to best friend’s smoking recommendation) among female students (8.2% in 2005, 4.4% in 2013), which was higher than that among male students (8.6% in 2005, 7.4% in 2013), which corroborates the above claims [31].

### Cohort effect

Before the admission cohort in 2009 (girls) and 2010 (boys), the cohort effect showed a pattern of initial decrease followed by an increase. However, a decrease was observed in both males and females after that point. Reinforcement of tobacco control policies and negative social perspective on smoking could affect adolescents awaiting middle school admission, who are the largest group for first smoking experience. Tobacco control laws such as display of carcinogen on cigarette pack in 2007; enforcing bans on tobacco advertising, promotion, and sponsorship in 2011; and restriction of smoking in public spread the negative perspective on smoking socially. The 2009~2010 admission cohort is thought to be the first to be exposed to these social perspectives.

A systematic school-based smoking prevention program with budget in Korea started experimentally in 2001~2003 in 100 schools. However, this size was insufficient to cause a decrease in the national adolescent smoking rate. In 2010~2014, the school-based smoking prevention program was expanded to 10% of schools nationwide, which must have also affected nearby non-participating schools indirectly. Furthermore, the increase in tobacco price in 2015 led to the implementation of school-based smoking prevention programs in all schools in the nation [32]. Changes in social perspective on smoking along with a widespread school-based smoking prevention program in 2010 could have partially had a decreasing cohort effect starting in the admission cohort in 2010.

In summary, the APC effect that contributed to decreased lifetime smoking rate among female students relative to male students are as follows: 1) reduced magnitude of increase in high grade level due to age effect, 2) a consistent decrease coinciding with large magnitude of decrease in period effect between 2006~2016 and 3) short increase period of cohort effect (girls: 2005 to 2009 cohort groups, boys: 2004 to 2010 cohort groups) and decreased magnitude of increase (girls: 2.44 times, boys: 7.35 times). On the other hand, the period effect increased in male students in certain years (2007, 2011). This resulted in an inverted U-shape where the lifetime smoking rate among male students increased in 2007~2008 and 2011. Among female students, the lifetime smoking rate consistently decreased.

This study has the following limitations. First, the results were composed of self-reported smoking, which could have underestimated the lifetime smoking rate. However, the possibility of measurement error due to the age effect based on indicators of lifetime smoking rate characteristic at different grade levels within the same school admission cohort group can be raised. As a result, the measurement error could have affected the lifetime smoking rate secular trend among female students. Secondly, regarding the APC analysis in adults, the APC effects were evaluated every 5 or 10 years. However, the APC effect was evaluated every year in this study, which could have led to unstable results; the study on American adolescents also performed APC analysis annually and reached a stable result [14]. Also, the magnitude of change in smoking rate was not negligible with a 1 year increase in this study and lifetime smoking rate in each school’s admission cohort and each grade showed consistent patterns. Furthermore, since this study used data from a nationwide annual survey on 6000 students in different grades and different genders, it should not have been difficult to achieve stable results.

This study is significant since it studied cohort effects on smoking trends among Asian adolescents. Furthermore, this study suggested that an APC analysis method for smoking rate secular trend analysis was necessary. Moreover, previous studies on APC analysis have implications for cohort and period effects rather than age effects, since age effect was a non-modifiable factor in terms of public health, although the age effect is a significant factor that influences smoking rate. In this study, the age effect results took into consideration the indicator characteristics and the possibility of measurement error in the self-reported survey was evaluated, which differed from other previous studies.

## Conclusion

APC effects were present in lifetime smoking rates among both male and female students. There was a difference between APC effects of the two genders. As a result, this difference influenced the formation of separate secular trends in lifetime smoking rates in the two genders. Therefore, considering the effect of APC could help us understand the trend in smoking rates and the contextual factors that affect it.

## Supplementary information

**Additional file 1: Table S1.** Estimates from age-period-cohort models using the intrinsic estimator method.

## Data Availability

Data are available from the Korea Centers for Disease Control and Prevention after approval of use for Korean researchers.

## Abbreviations

APC: Age-period-cohort
KYRBS: Korean Youth Health Risk Behavior Web-based Survey
WHO: World Health Organization
