# Supplementary material for: Gender differential secular trend in lifetime smoking prevalence among adolescents: an age-period-cohort analysis
Source: BMC Public Health. 2019 Oct 25;19:1374. doi: 10.1186/s12889-019-7735-8 (PMC6815049; doi:10.1186/s12889-019-7735-8)
Supplement: Supplementary file 1 — Additional file 1: Table S1. Estimates from age-period-cohort models using the intrinsic estimator method. [file 12889_2019_7735_MOESM1_ESM.docx]

Table S1. Estimates from age-period-cohort models using the intrinsic estimator method

|  | Boys | | | Girls | | |
| --- | --- | --- | --- | --- | --- | --- |
| Variables | Coefficient | Standard error | P value | Coefficient | Standard error | P value |
| Intercept | -1.4178 | 0.0008 | <0.001 | -2.1371 | 0.0014 | <0.001 |
| Grade |  |  |  |  |  |  |
| 7^th^ | -0.7008 | 0.0012 |  | -0.5680 | 0.0017 |  |
| 8^th^ | -0.2427 | 0.0010 | <0.001 | -0.1713 | 0.0014 | <0.001 |
| 9^th^ | 0.0186 | 0.0009 | <0.001 | -0.0243 | 0.0013 | <0.001 |
| 10^th^ | 0.2406 | 0.0008 | <0.001 | 0.2038 | 0.0012 | <0.001 |
| 11^th^ | 0.3171 | 0.0008 | <0.001 | 0.2770 | 0.0012 | <0.001 |
| 12^th^ | 0.3672 | 0.0008 | <0.001 | 0.2828 | 0.0012 | <0.001 |
| Survey period |  |  |  |  |  |  |
| 2006 | 0.1818 | 0.0013 | <0.001 | 0.4483 | 0.0019 | <0.001 |
| 2007 | 0.1930 | 0.0013 | <0.001 | 0.4075 | 0.0018 | <0.001 |
| 2008 | 0.1921 | 0.0012 | <0.001 | 0.3813 | 0.0017 | <0.001 |
| 2009 | 0.1464 | 0.0012 | <0.001 | 0.3000 | 0.0017 | <0.001 |
| 2010 | 0.0580 | 0.0013 | <0.001 | 0.2063 | 0.0018 | <0.001 |
| 2011 | 0.0608 | 0.0013 | <0.001 | 0.1237 | 0.0019 | <0.001 |
| 2012 | -0.0164 | 0.0013 | <0.001 | 0.0160 | 0.0020 | <0.001 |
| 2013 | -0.1002 | 0.0014 | <0.001 | -0.2111 | 0.0022 | <0.001 |
| 2014 | -0.1190 | 0.0014 | <0.001 | -0.3086 | 0.0024 | <0.001 |
| 2015 | -0.1885 | 0.0015 | <0.001 | -0.4215 | 0.0026 | <0.001 |
| 2016 | -0.2323 | 0.0016 | <0.001 | -0.5293 | 0.0028 | <0.001 |
| 2017 | -0.1756 | 0.0018 | <0.001 | -0.4126 | 0.0032 | <0.001 |
| School admission cohort |  |  |  |  |  |  |
| 2001 | 0.0969 | 0.0026 | <0.001 | 0.2460 | 0.0035 | <0.001 |
| 2002 | 0.0804 | 0.0019 | <0.001 | 0.2443 | 0.0026 | <0.001 |
| 2003 | 0.0393 | 0.0017 | <0.001 | 0.1946 | 0.0023 | <0.001 |
| 2004 | 0.0344 | 0.0015 | <0.001 | 0.1365 | 0.0021 | <0.001 |
| 2005 | 0.0884 | 0.0014 | <0.001 | 0.1171 | 0.0021 | <0.001 |
| 2006 | 0.1294 | 0.0014 | <0.001 | 0.1291 | 0.0021 | <0.001 |
| 2007 | 0.1964 | 0.0015 | <0.001 | 0.2006 | 0.0022 | <0.001 |
| 2008 | 0.2317 | 0.0015 | <0.001 | 0.2657 | 0.0023 | <0.001 |
| 2009 | 0.2630 | 0.0016 | <0.001 | 0.2855 | 0.0025 | <0.001 |
| 2010 | 0.2888 | 0.0016 | <0.001 | 0.2321 | 0.0026 | <0.001 |
| 2011 | 0.2537 | 0.0017 | <0.001 | 0.2016 | 0.0028 | <0.001 |
| 2012 | 0.1628 | 0.0017 | <0.001 | 0.1071 | 0.0029 | <0.001 |
| 2013 | 0.0201 | 0.0020 | <0.001 | -0.0927 | 0.0034 | <0.001 |
| 2014 | -0.1191 | 0.0024 | <0.001 | -0.2062 | 0.0042 | <0.001 |
| 2015 | -0.3097 | 0.0033 | <0.001 | -0.3687 | 0.0056 | <0.001 |
| 2016 | -0.5266 | 0.0046 | <0.001 | -0.5987 | 0.0077 | <0.001 |
| 2017 | -0.9301 | 0.0100 | <0.001 | -1.0939 | 0.0169 | <0.001 |
